# Intestinal Antiseptics

**Published:** 1899-11-11

**Authors:** 


### INTESTINAL ANTISEPTICS.

A great deal has lately been written about the use of intestinal antiseptics. There is no doubt that, while the importance of intestinal toxicity as a cause of disease has secured for itself the attention of all physicians, the use of means for removing this condition has become a thoroughly well recognised detail, or even principle, of treatment. But to say this is by no means to admit that the mere impregnation of the intestinal contents with germicidal or germ inhibiting drugs has proved particularly successful, or that it has obtained any large degree of favour at the hands of practical physicians, great as may have been its success in individual cases. It is rather because we have lately enlarged our ideas as to what may be included in the term "intestinal antiseptics," and because in the treatment of septic or toxic conditions of the intestinal contents we have adopted the good surgical principle that the removal of a substance which can decompose is an even more antiseptic measure than its impregnation with antiseptic drugs, that intestinal antiseptics have obtained the position which they hold at present. Among the intestinal antiseptics which are now recommended are such substances as sulphurous acid, chlorine, carbolic acid, sulpho-carbolate of soda, menthol, salol, turpentine, benzoate of soda, &c., and there can be but little doubt that, if the particular substance suitable to the case in question happens to be employed, much good may in many cases be done by their use. One cannot but observe, however, that in surgery we have passed far beyond the stage of merely applying antiseptics to septic matters. To remove septic material, and to provide a free drainage when there is any doubt as to the complete cleansing of the part, are the key-notes of modern surgery in regard to the treatment of septic cases; and to attempt to control intestinal sepsis by the application of antiseptics to the intestinal contents, effectual as it may sometimes seem to be in certain cases, is but to turn the clock backwards and to revert to the position taken up by surgery 20 years ago. Medicine, however, has not so limited its functions, for, in addition to such drugs as are mentioned above, we find among the measures now included under the heading of "intestinal antiseptics," the use of such things as calomel, rhubarb, grey powder, aloes, saline purgatives, antiseptic enemata, and irrigation of the large intestines —measures which, however great their influence in directly preventing decomposition of the intestinal contents, undoubtedly have, as their primary action, the removal of these contents from the position in which they are a source of mischief. Thus medicine ranges itself alongside with surgery. In both it is admitted that the use of antiseptics exercises some influence over septic processes, and that for this purpose they are by all means to be employed, but in both it is sufficiently obvious that the most effective antiseptic measures are those which ensure that the materials on the decomposition of which the sepsis depends shall be removed. In the treatment of intestinal sepsis purgatives would seem to occupy much the same position as drainage does in the treatment of a septic abscess, while irrigation appears to play the same *rôle* in both. But in neither can the mere impregnation of the contents with antiseptics be looked upon as the highest art.

